# Nicotine Protects Kidney from Renal Ischemia/Reperfusion Injury through the Cholinergic Anti-Inflammatory Pathway

**DOI:** 10.1371/journal.pone.0000469

**Published:** 2007-05-23

**Authors:** Claude Sadis, Gwen Teske, Geurt Stokman, Carole Kubjak, Nike Claessen, Fabrice Moore, Patrizia Loi, Bilo Diallo, Luc Barvais, Michel Goldman, Sandrine Florquin, Alain Le Moine

**Affiliations:** 1 Institute for Medical Immunology, Université Libre de Bruxelles, Gosselies, Belgium; 2 Department of Pathology, Academic Medical Center, University of Amsterdam, Amsterdam, The Netherlands; 3 Biovallée, Gosselies, Belgium; 4 Department of Anesthesiology, Erasme Hospital, Université Libre de Bruxelles, Brussels, Belgium; University of California, San Francisco, United States of America

## Abstract

Kidney ischemia/reperfusion injury (I/R) is characterized by renal dysfunction and tubular damages resulting from an early activation of innate immunity. Recently, nicotine administration has been shown to be a powerful inhibitor of a variety of innate immune responses, including LPS-induced toxaemia. This cholinergic anti-inflammatory pathway acts via the α7 nicotinic acetylcholine receptor (α7nAChR). Herein, we tested the potential protective effect of nicotine administration in a mouse model of renal I/R injury induced by bilateral clamping of kidney arteries. Renal function, tubular damages and inflammatory response were compared between control animals and mice receiving nicotine at the time of ischemia. Nicotine pretreatment protected mice from renal dysfunction in a dose-dependent manner and through the α7nAChR, as attested by the absence of protection in α7nAChR-deficient mice. Additionally, nicotine significantly reduced tubular damages, prevented neutrophil infiltration and decreased productions of the CXC-chemokine KC, TNF-α and the proinflammatory high-mobility group box 1 protein. Reduced tubular damage in nicotine pre-treated mice was associated with a decrease in tubular cell apoptosis and proliferative response as attested by the reduction of caspase-3 and Ki67 positive cells, respectively. All together, these data highlight that nicotine exerts a protective anti-inflammatory effect during kidney I/R through the cholinergic α7nAChR pathway. In addition, this could provide an opportunity to overcome the effect of surgical cholinergic denervation during kidney transplantation.

## Introduction

Ischemia/reperfusion (I/R) injury is a major cause of acute renal failure occurring after hemorrhagic shock or major cardiovascular surgery [1]. Despite renal replacement therapies, I/R injury remains associated with a high morbidity and a mortality rate estimated between 40–80% for intensive care patients. In the context of renal transplantation, enhanced I/R injury is responsible for delayed graft function affecting the long-term transplant outcome [2]. I/R is associated with a large number of pathophysiological alterations resulting eventually in the destruction of the renal tissue. I/R injury is considered as an inflammatory process originally triggered by tissue oxygen starvation, mitochondrial dysfunction and ATP depletion [3]–[6]. Upon hypoxic injury, tubular epithelial cells (TECs) acquire a pro-inflammatory phenotype and start to release cytokines and chemokines. Early after reperfusion, a massive influx of neutrophils is observed in the damaged areas. These neutrophils exert a crucial role in the pathophysiology of I/R injury by the release of proteases and oxygen-derived radicals, amplifying renal injury [7]–[9]. In these conditions, TECs undergo necrosis and apoptosis. In turn, necrotic products activate innate immunity through Toll-like receptors (TLR) signalling pathways leading to an amplification of the inflammatory response.

Recently, a body of evidence demonstrated that innate immune responses can be efficiently regulated by vagus nerve, a concept referred as the cholinergic anti-inflammatory pathway [10], [11]. This regulatory pathway acts in an α7 nicotinic acetylcholine receptor (α7nAChR)-dependent manner [12]. Acetylcholine, which is released by stimulated vagus nerve and nicotine, an α7nAChR agonist, suppress TNF-α production by LPS-stimulated macrophages. The anti-inflammatory properties of the cholinergic pathway have been also described in different in vivo models such as LPS-induced toxaemia, Schwartzman reaction, pancreatitis and peritonitis [13]–[16]. On the opposite, vagotomy or α7nAChR deficiency dramatically enhances the sensitivity to endotoxin. One mechanism by which the cholinergic pathway mediates its anti-inflammatory effects is by controlling the release of high-mobility group box 1 protein (HMGB1) [16]. A role for HMGB1 has been recently described in the context of liver I/R [17], [18]. Known as a late mediator of endotoxic shock, HMGB1 acts simultaneously as a chemoattractant and activator of immature dendritic cells through TLR-2, TLR-4 and the receptor of advanced glycation end products (RAGE) [19], [20]. In the present study, we hypothesized that nicotine pretreatment could modulate renal I/R injury through the cholinergic anti-inflammatory pathway.

## Material and Methods

### Mice

Male C57BL/6 mice were bred in our specific pathogen-free animal facility. Heterozygous α7nAChoR (α7+/−) B6-129S7 mice [12] were purchased from the Jackson Laboratory (Bar Harbor, Maine) and bred in our animal facility. First generation of α7+/+ and α7−/− animals were distinguished through genotyping following JAX-protocol instructions. Ten to 15 weeks-old male animals were used for I/R experiments. All mice of compared groups were weight-matched. All animals received care in compliance with the Principles of Laboratory Animal Care formulated by the National Institute of Health (NIH publication No. 86-23, revised 1985) and protocols were approved by the local committee for animal welfare - Comité d'Ethique du Biopole de Charleroi, Université Libre de Bruxelles.

### Renal ischemia/reperfusion model

Renal ischemia-reperfusion injury was induced by a 35 minutes bilateral clamping of renal arteries as previously described [7]. Male mice were anesthetized through an intraperitoneal injection (80 µl/10 g weight) of a mixture containing fentanyl citrate 0.08 mg/ml, fluanisone 2.5 mg/ml (VetaPharma Limited) and midazolam 1.25 mg/ml (Roche). After a median abdominal incision, both renal arteries were clamped during 35 minutes with microaneuvrysm clamps. Throughout ischemic period, evidence of clamping was confirmed by visualizing dark colour of ischemic kidneys. After clamp removal, adequate restoration of blood flow was checked before abdominal closure. Muscle and skin were closed in two layers and sterile NaCl 0.9% (300 µl) was injected subcutaneously to restore a balanced fluid volume. Mice were kept on a warming tap (38°C) for the next 12 hours with food and water available. Sham-operated animals underwent the same surgical procedure without clamping and were sacrificed 1 day after surgery. Saline-treated animals received an intraperitoneal injection of sterile NaCl 0.9% 30 minutes before renal clamping. One single dose of nicotine (Sigma) diluted in sterile PBS (Phosphate-buffered saline, pH 7.2) was injected intraperitoneally 30 minutes before surgery in either sham-operated group or mice undergoing renal I/R. I/R animals were sacrificed at day 1, 3 and 7 after reperfusion and blood and kidneys were harvested.

### Plasma biochemical analysis

The recovery of renal function was determined by measuring creatinine in plasma samples obtained after intervention by enzyme reactions involving creatinase and using standard autoanalyzer methods by hospital research services of Academic Medical Center, Amsterdam [7].

### Histology

Renal tissue was fixed for 24 hours in 10% formalin and embedded in paraffin. Tubular damages were assessed on PAS-D stained 4 µm-thick sections by scoring tubular cell necrosis, tubular dilatation, cast deposition and brush border loss in 10 non-overlapping fields (×400 magnification) in the corticomedullary junction [7]. Injury was scored by a pathologist blinded for the groups on a 5-point scale: 0 = no damage, 1 = 10% of the corticomedullary junction injured, 2 = 10–25%, 3 = 25–50%, 4 = 50–75%, 5 = more than 75%.

### Preparation of renal tissue for cytokine measurements

Kidneys were homogenised in a buffer containing 4mM EDTA, 1% Triton-X, 1% Protease Inhibitor Cocktail (Sigma) with the MagNa Lyser (Roche). TNF-α and KC were measured using specific ELISA (R&D Systems) according to manufacturer's instruction. The detection limits were 31 pg/ml and 15 pg/ml for TNF-α and KC, respectively. Values were corrected for the amount of protein in kidney tissue using the Bio-Rad protein assay (Bio-Rad).

### Immunostainings for neutrophils, apoptosis and proliferation

Four µm paraffine sections were cut, deparaffinized and rehydrated. Endogenous peroxidase activity was first quenched by hydrogen peroxide 0.3% in methanol. For neutrophil staining, sections were digested with pepsine (Sigma) 0.25% in 0.1 M hydrochloric acid and non specific binding was blocked with Normal Goat Serum 5% in PBS (DakoCytomation). Sections were then incubated with FITC-labelled anti-mouse Ly-6G mAb (551459; BD Biosciences), followed by an incubation with a rabbit anti-FITC antibody (DakoCytomation) and finally with PowerVision HRP-conjugated goat anti-Rabbit IgG solution (ImmunoVision Technologies, Co). Coloration was revealed using 1% DAB (Sigma-Aldrich) with 1% hydrogen peroxide in 0.05 M TRIS-HCl. For macrophage, apoptose and proliferation stainings procedures were essentially the same except that slides were cooked in 0.1 M citrate buffer. For macrophage staining, slides were incubated with rat anti-mouse F4/80 IgG2b mAb (MCA497R; Serotec), rabbit anti-rat biotin (DakoCytomation) and finally with streptavidin-ABC solution (DakoCytomation). For apoptose, sections were incubated overnight (4°C) with Cleaved Caspase-3 Antibody (Asp175; Cell Signaling) and for proliferation, sections were incubated overnight (4°C) with Rabbit Monoclonal anti-Ki67 (RM-9106; Lab Vision). Then, all sections were incubated with Power Vision Poly-HRP-Anti-Rabbit (Immunologics) and were developed using DAB plus hydrogen peroxide in 0.05 M TRIS-HCl as described before.

Positive cells were counted on 10 non-overlapping fields in the corticomedullary junction (×400 magnification) [7].

### HMGB1 detection by western blot

Western blots were performed on cytoplasmic extracts from renal cells. Kidneys were homogenized at 4°C in a lysis buffer containing 10 mM HEPES (pH 7.9), 1.5 mM MgCl2, 0.1% Igepal (INC Biochemics) mixed with 1% Protease Inhibitor Cocktail (Sigma). After 30 minutes of incubation at 4°C and 5 min of centrifugation at 7500 rpm (4°C), supernatants containing cytoplasmic proteins were separated from the pellet and stored at −20°C. Cytoplasmic extracts (100 µg protein/extract measured by Bio-Rad protein assay) were separated by electrophoresis on SDS-Polyacrylamide gel and transferred on PVDF membranes (Hybond-P). After blocking with 5% non fat dry milk in TRIS-buffered saline containing 0.1% Tween (TBS-T), membranes were first incubated with Rabbit polyclonal Anti-HMGB-1 (ab11972; Abcam) and with HRP-conjugated Anti-rabbit IgG (NA934VS; Amersham Biosciences). Immunoreactive bands were revealed with ECL Advance Western Blotting Detection Kit (Amersham Biosciences) and visualised with ChemiDoc ™ XRS System (Bio-Rad). Bands were quantified with Quantity One® software.

### Statistical analysis

Data are expressed as means+/−SEM. Non parametric Mann-Whitney two-tailed test was used to compare experimental groups and a p value <0.05 was considered as statistically significant.

## Results

### Effect of nicotine administration on ischemia/reperfusion injury and renal dysfunction

**Figure 1:**
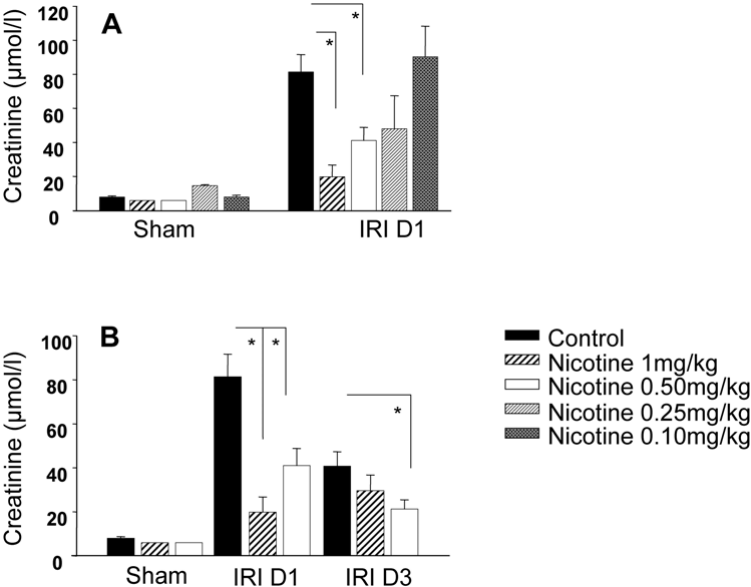
Nicotine protects renal function after I/R in a dose-dependent manner. A dose range from 0.1 to 1 mg/kg body weight of nicotine has been administrated intraperitoneally 30 minutes before the induction of renal ischemia (A). Only 0.5 mg/kg (n = 12) and 1 mg/kg (n = 15) of nicotine protects renal function 1 day after renal I/R. Doses lower than 0.5 mg/kg such as 0.25 mg/kg (n = 6) and 0.1 mg/kg (n = 7) were not protective. Data are expressed as mean ± SEM, (*) p≤0.05. On panel B, serum creatinine levels were measured in sham-operated animals that received either saline solution (black bars, n = 8) or nicotine at the dose of either 0.5 mg/kg body weight (white bars, n = 6) or 1 mg/kg body weight (n = 6) and compared with saline-treated (black bars, n = 19 at day 1 and n = 15 at day 3) or nicotine treated animals at doses of 0.5 mg/kg (n = 12 at day 1 and n = 15 at day 3) or 1 mg/kg (n = 16 at day 1 and n = 12 at day 3) that underwent renal I/R. Blood samples were harvested 1 and 3 days after reperfusion. Results are representative of three independent experiments. Data are expressed as mean ± SEM, (*) p≤0.05.

**Figure 2:**
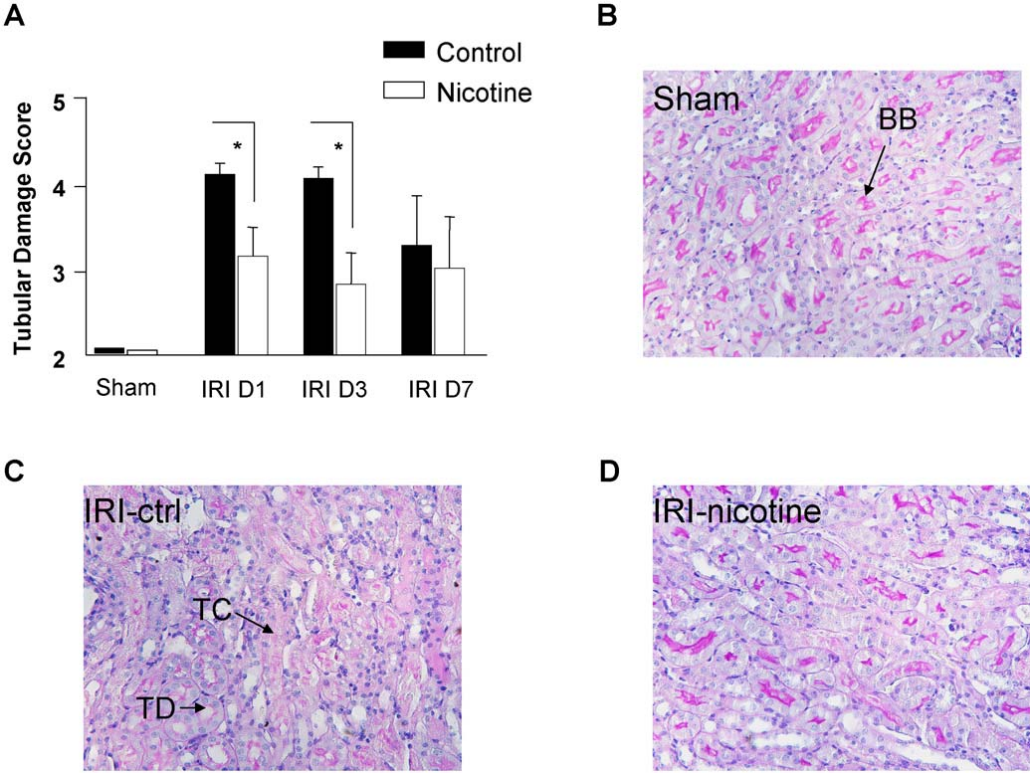
Nicotine controls tubular damage after renal I/R. Tubular damage score (A) is shown in sham-operated animals (n = 7 in each group), 1 and 3 days after I/R in either saline-treated (black bars, n = 17 and n = 14 at day 1 and 3, respectively) or nicotine-treated animals (white bars, n = 15 and n = 13 at day 1 and 3, respectively). Histology of the corticomedullary junction 1 day after reperfusion from a sham-operated mouse (B, referred as sham), a saline-treated mouse that underwent I/R (C, referred as IRI-CTRL) and a nicotine-treated mouse (D, referred as IRI-nicotine), PAS staining, original magnification ×40. The PAS positive brush border (BB) is shown in panel B. Tubular casts (TC) and tubular dilatations (TD) are visible in panel C. Similar results were obtained in two separate experiments. Data are expressed as mean ± SEM, (*) p≤0.05.

We tested the effect of nicotine administration on renal I/R injury in wild-type male C57BL/6 mice. A dose range from 0.1 to 1.0 mg/kg body weight was injected intraperitoneally 30 minutes before ischemia. Nicotine administration at doses varying from 0.1 mg/kg to 1 mg/kg did not affect renal function in sham-operated group (figure 1). As expected, saline-treated animals rapidly worsened renal function after I/R, as attested by increased serum levels of creatinine. Nicotine pretreatment protects renal function in a dose-dependent manner as shown by serum creatinine levels after different doses varying from 1 mg/kg to 0.1 mg/kg (figure 1A). The protective effect of nicotine pretreatment at the dose of 1 mg/kg was no longer present 3 days after reperfusion but still observed with a dose of 0.5 mg/kg (figure 1B). Seven days after reperfusion, serum creatinine in control and nicotine pre-treated groups was comparable to baseline creatinine levels measured in sham animals (data not shown). Therefore, the dose of 0.5 mg/kg was used for the next experiments. Kidney histology from I/R saline-treated mice revealed significant degrees of cell necrosis, tubular dilatation, loss of brush border and cast deposition at the corticomedullary junction (figure 2), achieving a score of approximately 4 on a scale of 5, 1 day and 3 days after reperfusion. Nicotine administration significantly reduced these tubular damages 1 day and 3 days after reperfusion. Seven days after reperfusion, both groups displayed comparable residual levels of damages (figure 2).

### Nicotine reduces the production of inflammatory mediators after renal ischemia/reperfusion

**Figure 3:**
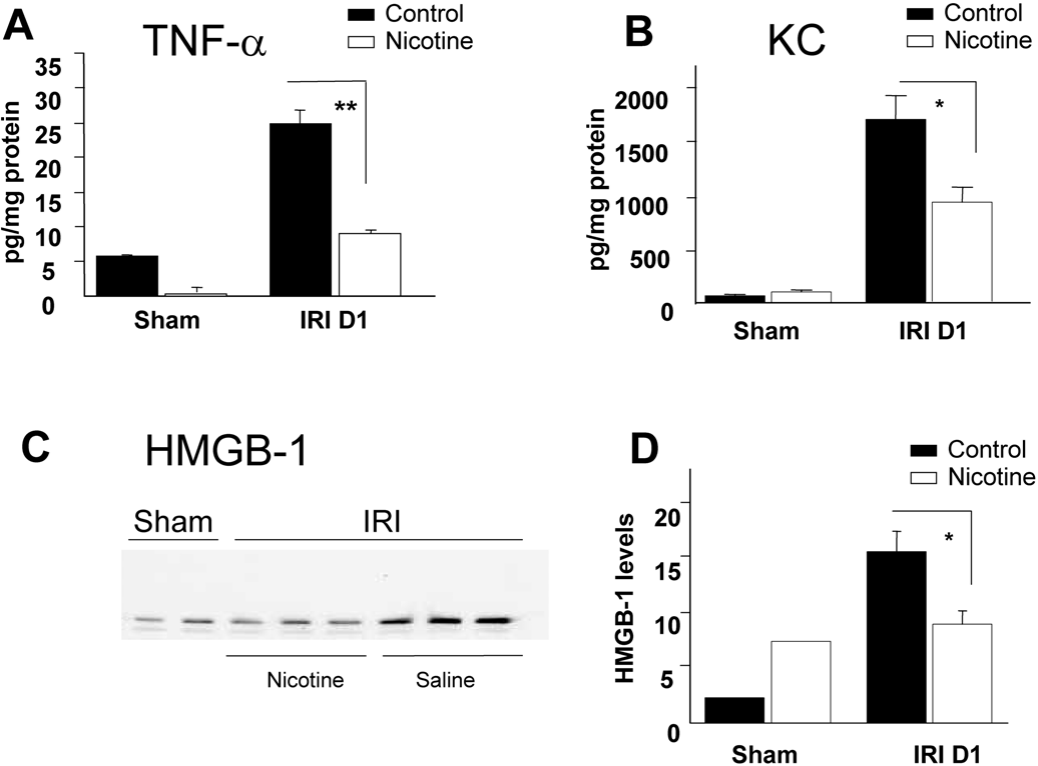
Nicotine down regulates TNF-α, KC and HMGB1 after renal I/R. The intra-renal production of TNF-α (A), KC (B) and HMGB1 (C and D) was measured in sham-operated animals (n = 6) and after I/R of either saline-treated animals (black bars, n = 8) or nicotine-treated animals (white bars, n = 8). TNF-α and KC levels were measured by ELISA in kidney homogenates harvested 24 hours after reperfusion. All samples were corrected for total protein amounts measured by Bio-Rad protein assay. HMGB1 specific blots (C) were performed on cytoplasmic renal extracts. From left to the right: saline treated sham-operated animals, nicotine-treated sham-operated animals, nicotine treated I/R animals and saline treated I/R animals. Blots are representative of 6 to 8 individual mice in each group. Quantification was assessed by the densitometry index (D). Similar results were observed in two separate experiments. Data are expressed as mean± SEM and (*) p≤0.05.

Nicotine has been identified as a potent posttranscriptional suppressor of pro-inflammatory cytokine production in sepsis [12], [16]. For this reason, we compared TNF-α, KC and HMGB1 levels in kidney homogenates from either nicotine-treated animals or saline-treated controls, 24 hours after reperfusion. Nicotine pretreatment almost completely suppressed TNF-α production observed in saline-treated animals and significantly decreased KC levels (Figure 3A and B). HMGB1 was measured by western blot. We first observed an increase in renal HMGB1 after I/R compared to sham animals. Nicotine administration significantly decreased HMGB-1 release compared to saline-treated animals (figure 3C and D).

**Figure 4:**
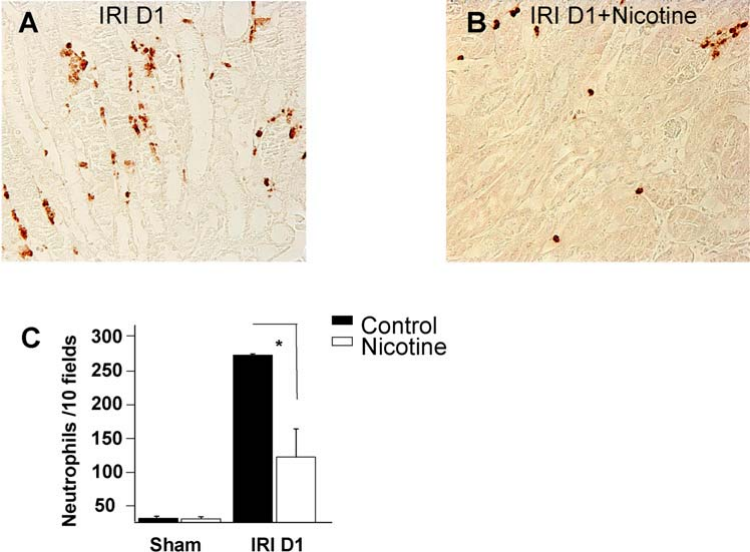
Nicotine pretreatment prevents neutrophil infiltration. LY-6G immunostaining was performed to assess neutrophil infiltration in the corticomedullary junction 24 hours after I/R (panel A, magnification ×40). Sham operated animals displayed no neutrophil (data not shown). Nicotine administration completely prevented neutrophil infiltration (panel B, magnification ×40). Quantification is represented in panel C. Black bars represent saline-treated animals and white bars nicotine-treated groups after either sham operation (n = 6 in each group) or I/R (n = 8 in each group). Data are expressed as mean ± SEM, (*) p≤0.05.

### Nicotine regulates neutrophil infiltrate after renal ischemia/reperfusion

To explore whether nicotine-mediated suppression of chemokine and cytokine production affects neutrophil influx, we performed immunostaining using the neutrophil-specific Ly-6G mAb. As expected, one day after reperfusion, a massive neutrophil infiltration was observed at the corticomedullary junction in saline-treated animals which was significantly reduced by nicotine pretreatment (figure 4).

### Nicotine administration decreases tubular epithelial cell apoptosis and proliferation after renal ischemia/reperfusion

**Figure 5:**
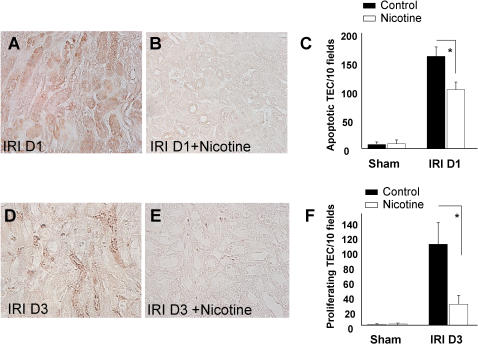
Nicotine reduces tubular epithelial cell apoptosis and subsequent proliferation. Immunostaining of cleaved caspase-3 (A, B) and Ki67 (D, E) were performed one day and 3 days after I/R, respectively. Panel A represents TEC apoptosis in saline-treated animals (magnification ×40) and panel B represents TEC apoptosis in nicotine pre-treated animals (magnification ×40). Graph C compares the quantification of apoptotic TECs in saline-treated (black bars) or nicotine-treated animals (white bars), either after sham operation (n = 6 in each group) or renal I/R (n = 7 in saline-treated group and n = 10 in nicotine pretreated group). Panels D and E compare Ki-67+ cells between saline-treated animals and nicotine-treated animals (magnification ×40). Graph F compares the quantification of proliferation 3 days after reperfusion (n = 5 in each group). Data are expressed as mean ± SEM, (*) p≤0.05.

Caspase activation and renal cell apoptosis are responsible for KC chemokine production promoting neutrophil infiltration in the kidney [21]. Because nicotine pretreatment modulates cytokine and chemokine release and reduces neutrophil infiltration, we measured its impact on TEC apoptosis and subsequent proliferation. The cleaved caspase-3 immunostaining showed a significant decrease of apoptotic TECs in the renal corticomedullary junction of nicotine pretreated animals compared to saline-treated animals 24 hours after reperfusion (Figure 5). Ki67 immunostaining, a nuclear protein expressed during G1-, S-, M- and G-2 phases of the cell cycle, was performed 3 days after reperfusion to evaluate TEC proliferation. In line with the relative preservation of the renal parenchyma in mice pretreated with nicotine, less TECs proliferation was observed after 3 days in this group compared to saline-treated group (Figure 5).

### Nicotine-mediated protection against ischemia/reperfusion injury is a7nAChoR-dependent

**Figure 6:**
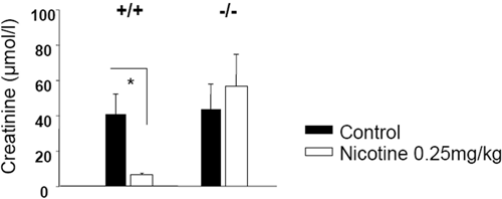
Nicotine-mediated protection against renal I/R is a7nAChoR-dependent. Serum creatinine levels one day after renal I/R were measured in the following groups from left to the right: saline-treated wild-type α7nAChoR sufficient animals (+/+, black bars, n = 10), nicotine-treated wild type α7nAChoR sufficient animals (+/+, white bars, n = 5), saline-treated α7nAChoR−/− animals (−/−, black bars, n = 8) and nicotine-treated α7nAChoR−/− animals. (−/−, white bars, n = 10). Nicotine was given at the dose of 0.25 mg/kg body weight. Data are expressed as mean ± SEM, (*) p≤0.05.

As attested by endotoxaemia experiments in genetically deficient mice, the α7 nicotinic receptor is a pivotal element in both naturally occurring and nicotine-mediated cholinergic anti-inflammatory pathway [12], [16]. Therefore, we wondered if nicotine-mediated protection against renal I/R was also α7nAChoR-dependent. Because α7nACho-subunit deficient (α7nAChoR−/−) mice are in another background, we first determined the protective dose of nicotine in control littermates (0.25 mg/kg body weight, data not shown). Then, we performed comparative renal I/R experiments in either control littermates (referred as +/+) or α7nAChoR−/− mice with and without nicotine pretreatment. Although nicotine pretreatment protected control littermates from renal I/R injury, this treatment was totally inefficient in α7nAChoR−/− animals (Figure 6).

## Discussion

Our data provide evidence that nicotine, an anti-inflammatory cholinergic agonist, protects renal function and controls tubular damages after I/R. In the I/R model as in other settings, nicotine most probably acts as a powerful anti-inflammatory drug by targeting multiple factors. Neutrophil infiltration was massively suppressed by nicotine pretreatment as attested by the Ly-6G immunostaining. This can be, in part, explained by the important reduction of KC chemokine release we observed and by other non-mutually exclusive mechanisms that were not investigated here. Indeed, Tracey et al. showed that nicotine down-regulates adhesion molecule expression such as V-CAM, I-CAM and E-selectin on endothelial cells through an α7nAChoR-dependent mechanism [15]. Moreover, nicotine decreases CD44 expression on endothelial cells [22], a molecule which is also involved in neutrophil recruitment as shown by mouse renal I/R experiments in which anti-CD44 treatment prevents neutrophil infiltration and I/R injury [8]. In contrast, we observed that nicotine does not interfere with renal macrophage infiltration (data not shown). It has been shown that nicotine-treated macrophages lose the ability to release proinflammatory cytokines, yet keep their capacity to produce IL-10 [23], therefore nicotine-treated residual macrophages could play a role in the post-reperfusion healing process. This hypothetical point remains to be investigated. In a comparable way to what was described for nicotine in the LPS-induced toxaemia, we also observed a down- regulation of renal levels of TNF-α after I/R. In parallel, HMGB1, another powerful proinflammatory factor, is up-regulated after renal I/R and down regulated by nicotine pretreatment as described in other experimental models [23], [24]. HMGB-1 has been recently identified as a potent pro-inflammatory mediator in sepsis and hepatic I/R injury [16], [17], [25]. During severe inflammation, HMGB-1 is translocated from the nucleus into the cytoplasm and released actively by macrophages or passively by necrotic cells [23], [24]. In a mouse model of sepsis, Tracey et al. showed that nicotine improves survival through the decrease of HMGB-1 released by activated macrophages still in an α7nAChR-dependent manner [16]. During hepatic I/R, a high HMGB-1 level promotes hepatic injury by NF-κB activation through TLR-4 binding. It has been suggested that HMGB-1 acts as an ‘alarmin’ and amplifies inflammatory response by binding TLR-4, TLR-2 and RAGE receptors [19]. This leads to the maturation of dendritic cells which might be deleterious in transplantation because of the sensitization of transplant recipient T cells. Consistent with these observations, Leemans et al. observed that TLR-2 expressed on TECs is critically involved in renal I/R injury by promoting cytokine release, neutrophil infiltration and apoptosis [7]. The role played by TLR4 in this setting is still unknown.

TEC apoptosis is recurrent in renal I/R injury, and is considered as a proinflammatory event per se [21]. In our study, TEC apoptosis was clearly diminished by nicotine pretreatment as well as the subsequent TEC proliferation. There are at least two reasons for this observation. First, this might result from a lesser inflammatory environment by nicotine-mediated suppression of both TNF-α and neutrophil infiltration. Second, nicotine has been described to have anti-apoptotic properties [26]–[28]. In turn, a reduced number of apoptotic TECs could jam the inflammatory cascade. Indeed, apoptotic cells have been shown to enable HMGB-1 release by activated macrophages in a sepsis model [25]. Therefore, the HMGB-1 down regulation we observed in nicotine-treated animals could also be explained by a reduction of TEC apoptosis.

Chronic administration of nicotine has been described to be nephrotoxic in the rat [29]. Indeed, nicotine accumulates preferentially in the kidney, where it creates an imbalance between the release of lipid peroxidation products and the endogenous anti-oxidant activity [29]. We also observed that oral administration of nicotine, started 4 days before the induction of renal ischemia up to the sacrifice, following the protocol of van Westerloo [14] increased renal dysfunction in our I/R model (data not shown). In contrast, administration of 0.5 mg/kg in one-shot, 30 minutes before renal ischemia, a dose similar to that used by others in sepsis [16], was protective. Of note, the dose of 1 mg/kg in one-shot did not afford protection 3 days after I/R, suggesting a late toxic effect after reperfusion. Despite a narrow range between therapeutic and toxic doses, the clinical use of nicotine is considered as relatively safe [30]. In this context, we also tested another nAChoR agonist, the 1,1-dimethyl-4-phenyl-L-pioperazinium-iodide (DMPP). In a liver I/R model, DMPP has been shown to significantly decrease plasma-ALT and cytokines 3 hours after reperfusion I/R but was ineffective 24 h of reperfusion [31]. In our model, DMPP also protected from renal I/R injury but virtually no advantage was observed compared with nicotine (data not shown).

The absence of α7nAChoR abolished the protective effect of nicotine pretreatment. This observation is in line with others showing that the integrity of the cholinergic anti-inflammatory pathway requires the α7nAChoR expression. Whether nicotine-mediated protection results from a direct interaction with tubular epithelial cells or from an indirect effect on innate immune response or both, remains to be determined. This suggests that α7nAChoR-specific agonists could be useful for preventing acute renal failure in several clinical settings.

In summary, our data demonstrate that nicotine administration protects renal function and limits tubular damage after I/R through an α7nAChR-dependent regulation of innate immune response. This suggests that nicotinic agonists should be considered for the prevention of acute renal failure occurring in major cardiovascular surgery and renal transplantation.

## References

[1] Gill N, Nally JV, Fatica RA (2005). Renal failure secondary to acute tubular necrosis: epidemiology, diagnosis, and management.. Chest.

[2] Joosten SA, Sijpkens YW, van Kooten C, Paul LC (2005). Chronic renal allograft rejection: pathophysiologic considerations.. Kidney Int.

[3] Bonventre JV, Zuk A (2004). Ischemic acute renal failure: an inflammatory disease?. Kidney Int.

[4] Boros P, Bromberg JS (2006). New cellular and molecular immune pathways in ischemia/reperfusion injury.. Am J Transplant.

[5] Lien YH, Lai LW, Silva AL (2003). Pathogenesis of renal ischemia/reperfusion injury: lessons from knockout mice.. Life Sci.

[6] Lieberthal W, Nigam SK (2000). Acute renal failure. II. Experimental models of acute renal failure: imperfect but indispensable.. Am J Physiol Renal Physiol.

[7] Leemans JC, Stokman G, Claessen N, Rouschop KM, Teske GJ (2005). Renal-associated TLR2 mediates ischemia/reperfusion injury in the kidney.. J Clin Invest.

[8] Rouschop KM, Roelofs JJ, Claessen N, da Costa MP, Zwaginga JJ (2005). Protection against renal ischemia reperfusion injury by CD44 disruption.. J Am Soc Nephrol.

[9] Tracey KJ (2007). Physiology and immunology of the cholinergic antiinflammatory pathway.. J Clin Invest.

[10] Metz CN, Tracey KJ (2005). It takes nerve to dampen inflammation.. Nat Immunol.

[11] Tracey KJ (2002). The inflammatory reflex.. Nature.

[12] Wang H, Yu M, Ochani M, Amella CA, Tanovic M (2003). Nicotinic acetylcholine receptor alpha7 subunit is an essential regulator of inflammation.. Nature.

[13] van Westerloo DJ, Giebelen IA, Florquin S, Bruno MJ, Larosa GJ (2006). The vagus nerve and nicotinic receptors modulate experimental pancreatitis severity in mice.. Gastroenterology.

[14] van Westerloo DJ, Giebelen IA, Florquin S, Daalhuisen J, Bruno MJ (2005). The cholinergic anti-inflammatory pathway regulates the host response during septic peritonitis.. J Infect Dis.

[15] Saeed RW, Varma S, Peng-Nemeroff T, Sherry B, Balakhaneh D (2005). Cholinergic stimulation blocks endothelial cell activation and leukocyte recruitment during inflammation.. J Exp Med.

[16] Wang H, Liao H, Ochani M, Justiniani M, Lin X (2004). Cholinergic agonists inhibit HMGB1 release and improve survival in experimental sepsis.. Nat Med.

[17] Tsung A, Sahai R, Tanaka H, Nakao A, Fink MP (2005). The nuclear factor HMGB1 mediates hepatic injury after murine liver ischemia-reperfusion.. J Exp Med.

[18] Izuishi K, Tsung A, Jeyabalan G, Critchlow ND, Li J (2006). Cutting edge: high-mobility group box 1 preconditioning protects against liver ischemia-reperfusion injury.. J Immunol.

[19] Yang D, Chen Q, Yang H, Tracey KJ, Bustin M (2006). High mobility group box-1 (HMGB1) protein induces the migration and activation of human dendritic cells and acts as an alarmin.. J Leukoc Biol.

[20] Yu M, Wang H, Ding A, Golenbock DT, Latz E (2006). HMGB1 signals through toll-like receptor (TLR) 4 and TLR2.. Shock.

[21] Daemen MA, de Vries B, van't Veer C, Wolfs TG, Buurman WA (2001). Apoptosis and chemokine induction after renal ischemia-reperfusion.. Transplantation.

[22] Serobyan N, Schraufstatter IU, Strongin A, Khaldoyanidi SK (2005). Nicotinic acetylcholine receptor-mediated stimulation of endothelial cells results in the arrest of haematopoietic progenitor cells on endothelium.. Br J Haematol.

[23] Ulloa L (2005). The vagus nerve and the nicotinic anti-inflammatory pathway.. Nat Rev Drug Discov.

[24] Lotze MT, Tracey KJ (2005). High-mobility group box 1 protein (HMGB1): nuclear weapon in the immune arsenal.. Nat Rev Immunol.

[25] Qin S, Wang H, Yuan R, Li H, Ochani M (2006). Role of HMGB1 in apoptosis-mediated sepsis lethality.. J Exp Med.

[26] Mai H, May WS, Gao F, Jin Z, Deng X (2003). A functional role for nicotine in Bcl2 phosphorylation and suppression of apoptosis.. J Biol Chem.

[27] Toborek M, Son KW, Pudelko A, King-Pospisil K, Wylegala E (2006). ERK 1/2 signaling pathway is involved in nicotine-mediated neuroprotection in spinal cord neurons.. J Cell Biochem.

[28] Dasgupta P, Kinkade R, Joshi B, Decook C, Haura E (2006). Nicotine inhibits apoptosis induced by chemotherapeutic drugs by up-regulating XIAP and survivin.. Proc Natl Acad Sci USA.

[29] Sener G, Sehirli O, Ipci Y, Cetinel S, Cikler E (2005). Protective effects of taurine against nicotine-induced oxidative damage of rat urinary bladder and kidney.. Pharmacology.

[30] Thomas GA, Rhodes J, Ingram JR (2005). Mechanisms of disease: nicotine–a review of its actions in the context of gastrointestinal disease.. Nat Clin Pract Gastroenterol Hepatol.

[31] Crockett ET, Galligan JJ, Uhal BD, Harkema J, Roth R (2006). Protection of early phase hepatic ischemia-reperfusion injury by cholinergic agonists.. BMC Clin Pathol.

